# Colloquia De Omnibus Rebus

**Published:** 1852-12

**Authors:** 


### COLLOQUIA DE OMNIBUS REBUS.

### Coll. V.—De remediis Novis, specifis, diabeticis, etc.

The introduction of dramatic composition into medical literature, is somewhat of a novelty — an *innovation* — but it has, at least, the advantage of serving to relieve the dull uniformity of a style which generally characterizes the contributions to a medical Journal. The reader, at all events, will not be likely to take exceptions to the following colloquy, nor to others of the same sort, when he learns that it is taken from the Edinburgh Monthly Journal of Medical Sciences, and that the *Dramatis Personæ* are as follows:

| | |
|---|---|
| *Obstetricus*, . . . . | Prof. Simpson. |
| *Chirurgus*, . . . . . | Prof. Syme. |
| *Medicus*, . . . . . | Prof. Christison. |
| *Physiologus*, . . . . | Prof. Bennett. |
| *Chemicus*, . . . . | Prof. Maclagan. |
| *Editor*, . . . . . | Dr. Wm. Robertson. |

Ed. Buffalo Med. Journal.

*Obstetricus* (to *Chirurgus.*) Might a friend venture to inquire what has disturbed your equanimity this evening?

*Chirurgus.* Even yours would have been unsettled by the gentleman's story, who drove from my door as you arrived.

*Chemicus.* A tall, handsome, young fellow. I wondered to see him leaving your hospitable gate at such an hour.

*Chirurgus.* He is not in condition to enjoy hospitality, and came here for a very different purpose. He is one of the

Victims of Mercury. Passing through Edinburgh with a mercurial sore-throat, a pocketful of mercurial prescriptions, and a mercurial belt, he felt uneasy traveling with three such unsafe companions, and came to see what I thought of him and them.

*Chemicus.* He would be surprised to learn that the root of his misfortune lay in his belt and recipes, and not in his throat.

*Chirurgus.* Very possibly. But I have not yet told you all. Led by incidental circumstances, he had been for some time indulging freely in wine and wassail, and living a life of hard exercise and constant exposure. On expressing my wonder at this, he told me, to my consternation, that the London surgeon, who advised him to poison himself with mercury, had not put him on his guard, or under any rule or restriction, as to diet or regimen. You may judge what reason I had for appearing discomposed.

*Chemicus.* The traveler has cause to thank his stars and his constitution of "oak and triple brass," that he had not bid adieu to his nose and palate at least. What a fearful amount of misery must arise from the wagon-loads of mercurial pills and potions which are administered in London to all sorts of weak and scrofulous victims of venereal disease! It is a subject of painful reflection to every mind not proof against every humane consideration.

*Chirurgus.* The *Athenæum* tells us the other day, that medical men "have a vested interest in fever and cholera; their estate consists in the foul places, the bad drains, the putrid heaps of the city grave-yards." If this opinion, which is doubtless founded on acquaintance with the sentiments of the author's medical friends—should fairly represent the tone of metropolitan medical ethics, it would be unreasonable to expect the abandonment of the mercurial treatment of syphilis. But we must hope that things are not quite so bad as might appear from the *Athenæum.* In every medical community there must be numbers of professional men who are not so blinded by the pursuit of gain as to have their eyes shut to the truth, because it may affect their pockets. There are even some bright exceptions to the dogmatic mercurialism of London surgery.

*Medicus.* Do you mean to tell us, that, after what has been done and written about syphilis and mercury during the last forty years, a non-mercurialist is still the exception in London practice?

*Chirurgus.* Certainly. Have we not perpetual proof of this in the contents of the London Journals, and in such living illustrations as my belted traveler—whose case, I can assure you, is by no means a solitary one in my observation.

*Medicus.* This is deplorable. When I first went to London, in 1820, satisfied by frequent experience in our Infirmary here, of the soundness of the non-mercurial doctrine, first propounded by the medical officers of the army, and then systematized and powerfully advocated by Dr. John Thomson, I was shocked to find, as pupil of one of the great metropolitan hospitals, its "foul-ward" patients salivating, many of them for the second, third, or fourth time, and its surgeons ignorant or regardless of the glorious victory over mercury gained by our army surgeons, and conclusively followed up in the North. Returning thither in 1838, I expected to encounter truth at last in the ascendant; but in vain. After the lapse of eighteen years there were the same wards, the same fœtid atmosphere, the same mercurial victims—other surgeons, but the same ideas. Is it possible that fourteen years more have wrought no decay in that old donjon keep of prejudice?

*Physiologus.* I can add my testimony that matters were in the same state in 1833, having found in its attics the same sort of patients, and spit-boxes, and atmosphere, and notions that year, while a pupil, as you did in 1820.

*Obstetricus.* When *Chemicus* and I accompanied the late Mr. Barnsby Cooper at his visit in Guy's Hospital, in 1836, we ascertained that every surgical patient in the hospital was taking mercury in one shape or another; and there is no reason to suppose that matters are any better yet, so far as syphilis is concerned.

*Chirurgus.* The more need, then, for us to show the contrast; which the Managers of the Infirmary have just put it in our power to do. The great additions now made in the new buildings will afford ample accommodation for venereal patients, who for many years have been excluded from the hospital. We shall thus enable the student, as well as others, to learn from personal observation the truth of the principles, which have been so long taught and practiced in Edinburgh:—that "Hunterian chancres" and other primary affections may be cured by simple local treatment, without any mercury; and that in most secondary cases, mercury, instead of being an antidote for venereal infection, is another poison, and nothing else.

*Editor.* But would you consider so slight a matter as a Hunterian chancre a fit subject for hospital treatment?—it is such a trifle now under the non-mercurial method.

*Chirurgus.* The more occasion to prove to our unbelieving neighbors that it is so.

*Editor.* And where will you obtain in Edinburgh secondary cases of such severity as to instruct pupils or convince skeptical Southrons?

*Chirurgus.* Edinburgh can still supply a few of indigenous growth, thanks to one or two surviving home believers in the specific virtues of mercury against syphilis; and any want of native produce will be amply made up by arrivals from other parts still groaning under the mercurial curse.

*Editor.* To what do you ascribe so great a disregard of advancement in therapeutics as this dogged perseverance of our London brethren in the mercurial delusion?

*Chemicus.* To metropolitan indifference for improvement originating from without;—Roman contempt for everything barbarian.

*Physiologus.* Don't you think it may be rather referred to the prevalence there of a blind, degrading faith in Specifics, of which this mercury in the cure of syphilis has long been the chief?

*Medicus.* To both the one and the other concurrently, but at the bottom to an imperfect, unsound therapeutical education.

*Chemicus.* Why look farther than to metropolitan apathy toward "outside" improvement. For example, there has not been a single improvement of any importance made here in the treatment of diseases during the last five and twenty years that has been admitted into London practice, except tardily and imperfectly, if admitted at all.

*Medicus.* That is a bold proposition, yet true, and which, I doubt not, you can substantiate, if it be called in question. It may well rouse our metropolitan friends to serious reflection. But meanwhile, look a little beyond this state of things, and I think you will find its origin to be mainly a radical defect of tuition in therapeutics.

*Chemicus.* It was a marvelous step backward, when in 1850 the whole Boards of medical education in London, by incomprehensible common consent, reduced their requirements in materia medica to a course of lectures of three months.

*Medicus.* A heavy blow and discouragement truly to therapeutics. And more than this:—it is a proof to me that the nature and scope of therapeutics have not yet been duly appreciated in the London schools, or by the board of education there.

Is it possible to estimate too highly the importance of this branch of medical science? What is the ultimate object of medicine but the cure of diseases? What then ought to be the ultimate object of all medical education, if it be not the knowledge of the means of cure? To what purpose should we teach anatomy, physiology, chemistry,—why pathology and diagnosis,—if we did not possess remedies, medical and surgical, which we could put into the hands of students when so instructed? But fortunately we do possess them,—indeed in too lavish profusion. And the best of them are hard to obtain, difficult to know, variable in quality, puzzling to select, nice to prepare, but above all most wonderful in action,—energetic, multifarious, complex, versatile, and singularly influenced by co-operating circumstances.

The ancients knew all this: Therapeutics, indeed, with semeiology, constituted almost their whole circle of medical science. The early modern physicians knew it also: Witness Matthioli's great folio *Commentationes*, which went through eleven editions during half the sixteenth century. Alston, the first British professor of materia medica, knew it. He stated it in this University in 1738, with a course of lectures of six months in duration, and I have never heard that either professor or student has since found the period too long. In all great medical schools of the present day, except one, the same opinion has prevailed. In Britain, under the united name of Materia Medica, on the Continent under the separate heads of Pharmacy and Therapeutics, the means of curing diseases are taught in just equilibrium with the other branches of medicine. In London alone has it entered into the understanding of man to conceive that pharmacy, therapeutics, diet, and regimen may be mastered by a student in sixty lectures. When, indeed, University, College, and afterward King's College, were founded on the model of that of Edinburgh, an attempt was made to place the materia medica on a satisfactory footing, and other London schools followed the example. But after a twenty year's trial the attempt, it seems, has signally failed: and in 1850 both the London College of Surgeons and the Apothecaries' Company, reduced their requirements in materia medica to the old miserable standard.

*Chirurgus.* Possibly they thought that all which is at present positively ascertained on the subject may be taught in three months.

*Chemicus.* If professors of medicine and surgery were to teach only what is positively known in their several departments, few of them would require more time. It is the very uncertainty of materia medica, and especially of therapeutics,—the number of doubtful points to be discussed, the quantity of falsehood to be cleared up, the amount of fashionable humbug to be exposed, that entail the necessity of deliberate tuition.

*Medicus.* Exactly so. But unfortunately, in the London system there has long been no time left for anything but hasty tuition in this and some other equally important branches. The dominant influence of the College of Surgeons as an educational body,—the partial, narrow views of their Council, who now, as in time past, will look to nothing but anatomy and surgery as deserving of earnest attention,—have been the main cause of this. With the Council of the College, anatomy and surgery have been everything; at least everything else is little more than nothing. Even physiology and pathology by their regulations mere offsets or appendages to anatomy, and to be taught as branches of it,—a very natural error for a body composed entirely of hospital surgeons and lecturers on anatomy and surgery, and in which no other branch of medical science or art is represented. And as for the Apothecaries' Company, it is easy to see why they do not encourage the science which they ought peculiarly to foster; they cannot even yet overcome the old hallucinations that apprenticeship is education, and that a student, who is constantly handling drugs, must necessarily come to know all about them.

The consequences of all this might have been foreseen. What their directors undervalue, students do not prize. What the magnates of the profession do not cherish, the masses neglect. Therapeutics has ceased to be an object of inquiry, or is cultivated without method or principles. No one seems to care to improve our knowledge of old remedies. There is an incessant thirst for new ones. But these are sought for by the rule of chance; and not so much because they are needed for the purpose to which they are applied, and for which there is no want of acknowledged means; but apparently to satiate a morbid public craving for novelty, or to serve as a periodical invitation and advertisement. A wide-spreading empiricism broods over medicine, penetrating even into high places; and quackery of all kinds grows rank under its shade, pervading even the regular profession.

*Obstetricus.* You take a gloomy view of things. But the very magnitude of the evil will by and by work out its own reformation.

*Medicus.* It is not easy to avoid despondency, when one beholds, in relation to so essential a branch of medical science and practice, the ignorance of the profession, the advance of quackery, the sneers of the public, and the apathy of our medical rulers.

*Chemicus.* "Apropos des Charlatans," I see.

Treatment on Diabetes. Having attained something very like a true pathology of the disease,—having discovered that it is not a disorder of the kidneys but a depraved digestion,—and having ascertained the chemical composition of all the principal articles of man's food,—by theory it was at once inferred, that a number of old remedies in the shape of physic, and many new ones still proposed from time to time, may be allowed to sink into oblivion. By theory, too, we know that a peculiar regulation of the diet constitutes the only sound treatment; and we know also what articles compose that diet,—thus already making a great stride towards the cure. For, by the substitution of gluten-bread and cakes made of bran, butter, and eggs, for ordinary bread and other farinaceous food,—and by allowing such vegetables as spinage, cauliflower, brocoli, and cabbage which contain little or nothing capable of conversion into sugar, we have rendered a permanent nitrogenous diet practicable, which it was not before,—and so we effect sometimes a cure, and often a most material amendment, which may be maintained indefinitely by due dietetic observance.

*Obstetricus.* Have you seen any one recover entirely in that way?

*Medicus.* A gentleman of sixty-five recovered entirely three years ago, and continues well, unless he exceeds at table; and another of twenty-five, and a third, a boy of thirteen, are greatly improved,—the latter, indeed, might be thought in all respects well, except that the urine continues saccharine.

*Obstetricus.* Although we do not now know any medicine to improve this state of things by directly controlling the morbid peculiarity of digestion which constitutes the disease, who knows that theory may not soon direct us to one?

*Medicus.* It is much more likely to do so than empirical trial, that is, accident,—which has been hitherto followed as the main guide. Indee, know not but that it may have actually pointed out a remedy already. At least I have just received some very apposite information, which may interest you, relative to an entirely new remedy, derived strictly from theoretical considerations,—namely, the

Treatment of Diabetes by Rennet, which seems to promise well. Dr. Gray, of Glasgow, was lately induced to make trial of this substance by the following theoretical views. Diabetes consists in the process of digestion stopping at the conversion of other organic principles into sugar, which cannot be oxidated in the lungs, and is therefore thrown off as excrementitial by the kidneys. But rennet out of the body converts sugar into lactic acid, and it may therefore do so within the body likewise. Should such conversion take place, however, the disease will be brought to an end, if Liebig be right in his opinion, that lactic acid is one of the principles of the organic world which can support respiration, by becoming oxidated in the lungs. Resting on these views, Dr. Gray tried rennet in the case of a patient so much reduced by diabetes, of at least twelve months' standing, as to be unable to work. Dietetic treatment had been only of partial benefit. Medicines of various kinds had been of little use. The urine was copious, 1045 in density, and strongly saccharine. On the 30th of last July, a teaspoonful of rennet, prepared as for the dairy, was given thrice a day. In eight days the density of the urine was reduced to 1025, and it contained lactic acid, but only a trace of sugar. In twenty-five days the quantity was sixty-four ounces, the density 1022.5, and the sugar gone entirely. In six weeks the urine continued free of sugar; the man had gained weight considerably; his strength was such as to enable him to return to his employment; he thought himself in as good health as before his illness; and nevertheless he had been ten days on nearly his usual allowance of wheaten bread.

Now I am far from meaning to say, nor does Dr. Gray say, that rennet is thus proved to be a remedy for diabetes by its apparent success in a single case. But it is surely the most feasible remedy that has been proposed for many a day;—so feasible, that I hope many will give it at once a fair trial, which is his object in allowing me to give this brief notice of it to you all. Should it prove as successful in other hands as in his, we shall owe another therapeutic discovery to therapeutic theory.

*Obstetricus.* Were all inventors in the materia medica as well trained in therapeutics as Dr. Gray appears to have been, we should have fewer new remedies to deal with, and probably more good ones. It is certainly a striking confirmation of your criticism on London therapeutics, that, among the many new London remedies, not one has been announced for some years, which has stood the test of experiment elsewhere.

*Medicus.* A very natural consequence of the contempt manifested everywhere in London for therapeutic instruction. By the way I forgot to advert to a most extraordinary circumstance connected with the discountenancing of this branch of medical knowledge by the London boards of education,—viz., the complete and universal silence and submission with which their degrading regulations have been received. Not a single teacher has publicly uttered a single remonstrance. Not a journal has issued one word of criticism. Therapeutics, it seems, has not a patron in the whole metropolis. But enough of this for the present.

*Physiologus.* You mentioned a little ago that we had arrived at something like a sound pathology of diabetes, and that it seems to be a disease of digestion. But you are aware that this view may require revision, since the recent discoveries of Mr. Bernard, relative to the functions of the liver, by which he has proved that

SUGAR IS A NATURAL PRODUCT OF THE LIVER.

*Medicus.* That is possible. We do not yet see how the singular observations of Bernard are to affect the pathology of diabetes; but that they must have important bearings on it we can scarcely doubt. His inquiries have received too little attention in this country as yet. You have studied them carefully, and indeed have witnessed its leading experiments. Will you give us some account of them?

*Physiologus.* Within the last two years M. Bernard has brought forward a theory as to the production of sugar in the blood, which is supported by an amount of experimental proof that cannot be easily set aside. He admits that sugar may be formed in the process of digestion, and that a certain amount of it may, as the result of absorption from the alimentary canal, find its way into the blood. But he has shown that in man and animals of various orders, even so low down in the scale of creation as acephalous mollusca —if they are even fed entirely upon flesh—the blood from the hepatic vein invariably contains sugar. It is the result, however, of digestion of the food: for it disappears when an animal is starved, and it re-appears when the food is again given. He further observes, that sugar is found in the liver independently of the nature of the aliment. In dogs fed exclusively on animal food for several months, though he could find no sugar in the intestines or portal blood at its entrance into the liver, he always found it in the liver itself, and in the hepatic vein. In the spring of 1851 M. Bernard was good enough to perform the following experiment in my presence, during a visit I paid to Paris: A ligature was tied round the vena portæ where it enters the liver, and the dog was immediately killed by dividing the medulla oblongata. On opening the abdomen, the portal blood below the ligature, and blood from the hepatic vein, were immediately collected in separate glass vessels; and it was at once demonstrated, by applying the same test to both, that the latter contained sugar in abundance, but the former none. Sugar was also found in water in which a piece of the liver had been boiled in chips. Such an experiment seems decisive of the fact, that sugar is formed in the liver, and not conveyed to it with the blood through the vena portæ. Subsequently M. Bernard found that sugar is formed even by the fœtal liver; for he detected it in that organ both in mammals at different stages of intra-uterine life, and in birds before being hatched.

In all cases the sugar so formed presents the characters of grape-sugar. In all cases it is quickly decomposed on coming in contact with the blood and animal tissues. Hence, even in the livers of animals, it can be discovered only for a short time after death.

M. Bernard next discovered, that section of both pneumo-gastric nerves, as well as any violent shock to the nervous system, destroys the power of the liver to form sugar. The most interesting, however, of his observations, and that which bears most pointedly on the pathology of diabetes, is, that irritation of the root of the pneumo-gastric nerves in the fourth ventricle of the brain increases the formation of sugar in the liver, and causes it so to abound in the blood that is secreted with the urine; in short, this operation produces artificial diabetes. M. Bernard showed me this remarkable experiment. Having squeezed some urine from the bladder of a healthy rabbit, he proved that it did not contain sugar. He then passed a needle through the skull in such a way as to irritate the pneumo-gastric roots, and let the animal rest for an hour after the slight convulsions excited by the injury. Sugar was then found largely in its urine. On then killing the rabbit, it was found that the needle had wounded the intended part. I have since repeated this interesting experiment, with the same result; and so has my former assistant, Dr. Drummond: so that there can be no doubt of the fact.

*Medicus.* It has also been lately repeated with success in many trials by Dr. Schrader, as announced to the Royal Society of Sciences at Gottingen, in the beginning of the present year.

*Physiologus.* M. Bernard has since informed me of the results of his farther researches on this subject. He has now discovered, that, although section of the pneumo-gastric nerves destroys the formation of sugar in the liver, it is restored by artificially irritating their cut extremities; and that diabetes is produced exactly in the same manner as by irritating their origins in the brain. He was therefore led to conclude that the nervous action on the liver, necessary for the secretion of sugar, is not direct along the pneumo-gastrics, as he formerly supposed, but indirect, or reflex, through these nerves as incidents, the medulla oblongata as the center, and the spinal cord communicating with the solar ganglion as the excident channels of communication. And following out this theory, he likewise found that whenever the respiratory function is violently stimulated, sugar appears in the urine, and that whenever ether or chloroform is given, a temporary diabetes is occasioned. It follows that the formation of sugar by the liver is analogous to those kinds of secretion which are produced by reflex action through the agency of a sympathetic ganglion, and the influence of certain stimuli—such, for instance, as the secretion of saliva caused by the presence of sapid bodies in the mouth, where the sensitive and motor branches of the fifth pair operate in a reflex way through the agency of the submaxillary ganglion. In this case, stimulating the tongue is necessary to cause a flow of saliva; and in like manner, a certain stimulus of the lungs (normally by the air) is necessary to cause the formation of sugar by the liver. M. Bernard further supposes, that in the same way that the lungs thus act by reflex nervous influence on the liver, so does increased action of the liver act upon the kidney; consequently, that the sugar, produced in excess by one organ, is excreted by the other.

Such is the present state of the question. Various pathological considerations might be stated which seem to show that Bernard's liver theory of the origin of diabetes is as consistent with facts as the theory which ascribes it to disorder in the stomach. But further inquiry is necessary before we can positively settle the real cause of that very mysterious disease. Meanwhile, it is not easy as yet to see how the discoveries of Bernard will enable us to improve the treatment of diabetes, unless the well-known symptom of dryness of the skin, by exciting the lung to increased transpiration, be connected with the cause of the disorder, in which case diaphoretics, though they have been often used with some benefit, would be more strongly indicated. But I think something will be learnt on this head ere long.

*Editor.* Gentlemen, I must beg you to excuse me for breaking up this colloquy so soon. I must prepare for an early start to Rotterdam.

*Physiologus.* And I to Paris.

*Chemicus.* And I to the Doune of Rothiemurchus.

*Chirurgus* (*aside.*) And *Medicus*, *Obstetricus*, and I, to the top of The Cobbler.—*Edinburgh Monthly Journal.*

